# Molecular Profiling of Mouse Models of Loss or Gain of Function of the KCNT1 (Slack) Potassium Channel and Antisense Oligonucleotide Treatment

**DOI:** 10.3390/biom14111397

**Published:** 2024-11-02

**Authors:** Fangxu Sun, Huafeng Wang, Jing Wu, Imran H. Quraishi, Yalan Zhang, Maysam Pedram, Benbo Gao, Elizabeth A. Jonas, Viet Nguyen, Sijia Wu, Omar S. Mabrouk, Paymaan Jafar-nejad, Leonard K. Kaczmarek

**Affiliations:** 1 Biogen Inc., Cambridge, MA 02142, USAomar.mabrouk@biogen.com (O.S.M.); 2 Department of Pharmacology, Yale University School of Medicine, New Haven, CT 06520, USA; 3 Department of Neurology, Yale University School of Medicine, New Haven, CT 06520, USA; 4 Department of Internal Medicine, Yale School of Medicine, New Haven, CT 06520, USA; 5 Ionis Pharmaceuticals Inc., Carlsbad, CA 92010, USA; 6 Department of Cellular Molecular Physiology, Yale School of Medicine, New Haven, CT 06520, USA

**Keywords:** slack, KCNT1, K_Na_1.1, epilepsy, intellectual disability, EIMFS, mitochondria, proteomics

## Abstract

The potassium sodium-activated channel subtype T member 1 (*KCNT1*) gene encodes the Slack channel K_Na_1.1, which is expressed in neurons throughout the brain. Gain-of-function variants in *KCNT1* are associated with a spectrum of epilepsy syndromes, and mice carrying those variants exhibit a robust phenotype similar to that observed in patients. *Kcnt1* knockout (KO) mice, however, have a normal lifespan without any epileptic phenotype. To understand the molecular differences between these two models, we conducted a comprehensive proteomic analysis of the cerebral cortices of *Kcnt1* KO and *Kcnt1*^R455H/+^ mice, an animal model bearing a cytoplasmic C-terminal mutation homologous to a human R474H variant that results in EIMFS. The greatest change observed in *Kcnt1* KO mice compared to the wild-type mice was the increased expression of multiple proteins of the inner mitochondrial membrane. Electron microscopy studies of cortical mitochondria from *Kcnt1* KO mice further confirmed a significant increase in the density of mitochondrial cristae compared to that in wild-type mice. *Kcnt1* reduction by a murine-specific *Kcnt1* antisense oligonucleotide (ASO) in *Kcnt1*^R455H/+^ mice partially corrected the proteomic dysregulations in the disease model. The results support the hypothesis that ASO-mediated *KCNT1* reduction could be therapeutically useful in the treatment of *KCNT1* epilepsies.

## 1. Introduction

The *KCNT1* gene encodes a sodium-activated potassium channel (K_Na_ channel) termed Slack (also termed KCNT1, K_Na_1.1, or Slo2.2) that is widely expressed in the central nervous system [1,2,3,4,5]. Changes in the levels or activity of the Slack channel have a major consequence for neuronal excitability as well as learning and behavior in both mice and humans. For example, the loss of *Kcnt1* in young mice produces a complete loss of the most commonly studied forms of synaptic plasticity (long-term potentiation (LTP) and long-term depression (LTD)) [6] and disrupts cognitive flexibility in adult animals [7]. While it is not known if any humans have full loss of *KCNT1* expression, point mutations of the channel in humans give rise to multiple early-onset epilepsies associated with very severe intellectual disability. Two major conditions attributed to such point mutations are epilepsy of infancy with migrating focal seizures (EIMFS) and autosomal dominant nocturnal frontal lobe epilepsy (ADNFLE) [8,9,10].

In the *KCNT1* mutations that have been characterized in heterologous expression systems, the disease-causing mutations do not produce any loss of function but instead produce increases in the K_Na_ current with no change in the levels of Slack protein in the plasma membrane [11]. The increases in current result from changes in voltage sensitivity, Na^+^ sensitivity, and/or increased cooperative gating within clusters of channels in the plasma membrane [11,12]. One of the greatest increases in K_Na_ current has been found for *Kcnt1-R455H* (in rodent numbering) [11]. This mutation corresponds to the human mutation *KCNT1-R474H*, which causes EIMFS. In common with humans, a *Kcnt1-R455H* knock-in mouse model that is heterozygous for this mutation has seizures and persistent interictal spikes, but homozygous *Kcnt1-R455H* mice do not survive birth [13]. Characterization of *Kcnt1-R455H* mice has shown that this mutation increases K_Na_ currents and alters the intrinsic excitability of both the excitatory and inhibitory neurons of the cerebral cortex [14]. In addition, the mutation increases axon initial segment length and the expression of Na_V_ channel subunits in both types of neurons [14].

Slack is one of a subset of ion channels whose cytoplasmic domains interact directly with the mRNA-binding protein fragile X mental retardation protein (FMRP) [15,16,17,18,19,20,21,22,23]. Recent work has shown that the interaction with FMRP is dynamic, such that the activation of Slack channels triggers the initiation of the translation of a subset of neuronal mRNAs [23,24]. Moreover, the basal rate of translation is constitutively activated in cortical neurons of *Kcnt1-R455H* mice [24].

We carried out a proteomic analysis of *Kcnt1^−/−^* and *Kcnt^R455H/+^*mice and compared it to that of wild-type mice. In addition, recent approaches have begun to use antisense oligonucleotides targeted against *Kcnt1* to attempt to counteract the gain-of-function phenotype of the disease-causing mutations [25]. We therefore also used a murine-specific *Kcnt1* antisense oligonucleotide (ASO) to conduct a comprehensive proteomic analysis of *Kcnt1*^455H/+^ cortex tissue in mice receiving *Kcnt1* ASO treatment. Our findings indicate that loss of Slack results in changes in multiple cellular pathways including mitochondrial function. The *Kcnt^R455H/+^*mutation produced a different set of changes in the molecular profile, and *Kcnt1* ASO treatment of mice bearing this gain-of-function mutation restored some of these proteomic changes to the wild-type levels.

## 2. Materials and Methods

### 2.1. Animals

The *Kcnt1* KO [7,13,26] and *Kcnt1*^R455H/+^ [13] mice used in this study were characterized in previous publications. There were 10 animals in each group. All studies were carried out using mice that were 6–8 weeks of age. All experiments were performed in accordance with the NIH Guidelines for the Care and Use of Laboratory Animals and approved by Yale University’s Institutional Animal Care and Use Committee (IACUC).

### 2.2. Intracerebroventricular (ICV) Injections

*Kcnt1^R455H/+^* and wild-type mice at 6–8 weeks of age were treated with 500 µg of *KCNT1* ASO or control ASO via ICV injection. ASOs were administered to the right lateral ventricle. Analgesia was provided via local bupivacaine and subcutaneous buprenorphine and meloxicam per institutional guidelines. Mice were anesthetized and maintained with 1–2% isoflurane with a nose cone affixed to the stereotaxic instrument. A vertical incision was made over the scalp. A burr hole was drilled over the injection site. A 35-gauge sterile needle was advanced into the right lateral ventricle to coordinates of 0.3 mm anterior, 1.0 mm to the right, and −3.0 mm dorsal/ventral, relative to the bregma. Prior to ASO injections, adequate delivery to these coordinates was confirmed in a test animal via dye injection, which fully infused the ventricular system. A total of 10 µL of ASO or control solution was infused in each animal at a rate 20 nL per second. The burr hole was sealed with dental cement, the incision was sutured, and the animal was allowed to recover in a heated cage. After four weeks, animals were euthanized, and cortex tissue was collected and stored at −80 °C until further analysis.

### 2.3. Protein Extraction and Digestion

Mouse brain tissues were homogenized with FastPrep (MP Biomedicals, Santa Ana, CA, USA) in a lysis buffer of 8 M urea in 100 mM Tris-HCl, pH 7.4, with protease inhibitor (1 tablet/10 mL, Roche Diagnostics GmbH, Mannheim, Germany). After centrifuging, the supernatant was transferred to a new tube. The protein concentration was determined with a BCA protein assay. Disulfides on proteins were reduced with 15 mM DTT for 60 min at 37 °C and alkylated with 35 mM IAA at room temperature (RT) for 30 min in the dark. The samples were diluted with 50 mM ammonium bicarbonate to a final urea concentration of 1.5 M, followed by Lys-C (protein-to-protease ratio of 100:1 *w*/*w*, Wako, Osaka, Japan) digestion for 1 h at 37 °C and trypsin digestion (protein-to-protease ratio of 30:1 *w*/*w*, Promega, Fitchburg, WI, USA) overnight at 37 °C. The digestion was quenched by adding trifluoroacetic acid (TFA, Fisher Scientific, Waltham, MA, USA) to pH < 2. The peptides were desalted using tC18 Sep-Pak cartridge (Waters, Milford, MA, USA) and dried in a SpeedVac.

### 2.4. Peptide TMT Labeling and Fractionation

The peptides from all the samples were labeled with tandem mass tag (TMT) reagents (Thermo Scientific, Waltham, MA, USA) according to the manufacturer’s protocol. Peptides from wild-type, *Kcnt1^R455H/+^*, and *Kcnt1* KO mice were labeled with two sets of TMT-16plex, while peptides from wild-type and *Kcnt1^R455H/+^* mice treated with *Kcnt1* ASO or control ASO were labeled with two sets of TMT-18plex. Briefly, the peptides from each sample were dissolved in 50 µL of 100 mM ammonium bicarbonate buffer (pH 8.6), which was followed by adding 15 µL of TMT reagent that was dissolved in acetonitrile (ACN). The TMT labeling reaction continued for 60 min at room temperature with shaking and then was quenched by incubation with 10 µL of 5% hydroxylamine for 15 min. The labeled peptides from each set of TMT were mixed, and then the mixed sample was desalted using a tC18 Sep-Pak cartridge. The dried peptides were loaded onto a reverse-phase HPLC column (Waters), separated into 96 fractions using a 90 min gradient of 5–55% ACN containing 0.05% ammonium hydroxide (pH = 10), and then combined into 24 fractions. Each fraction was purified using the StageTip method before LC-MS/MS analysis.

### 2.5. LC-MS/MS Analysis

Peptides were dissolved in 0.1% TFA and loaded into a Thermo EASY-Spray C18 column with an EASY nLC 1200 (Thermo Scientific, Waltham, MA, USA). Peptides were first separated over a 120 min effective gradient at a flow rate of 275 nL/min and then detected using an Orbitrap Eclipse Tribrid mass spectrometer (Thermo Scientific, Waltham, MA, USA) with the SPS-MS3 method. A full MS scan (resolution: 120,000) was recorded with the Orbitrap at an automatic gain control (AGC) of 5 × 10^5^. The precursor ions with the top 10 highest intensities were fragmented using collision-induced dissociation (CID) with 35% normalized energy. Real-time search (RTS) was enabled for MS3 analysis. Selected ions were further fragmented under HCD at 45% NCE, and MS3 was acquired in the Orbitrap at a resolution of 50,000.

### 2.6. Database Searching, Data Filtering, and Bioinformatic Analysis

MaxQuant (version 2.1.3.0) was used to search the raw files against the database containing all mouse proteins downloaded from UniProt (Mus musculus) [27]. The search parameters included 20 ppm precursor mass tolerance; 20 ppm product ion mass tolerance; fully digested with trypsin; up to two missed cleavages; fixed modifications: carbamidomethylation of cysteine (+57.0214), TMT tag of lysine, and the peptide N-terminus (+304.2071); variable modifications: oxidation of methionine (+15.9949). The FDRs of peptide spectral matches (PSMs) and proteins were both filtered to <1%.

The protein data exported from MaxQuant were normalized to the median intensity of all proteins within each TMT set. The normalized intensity for each protein was then transformed to a relative ratio by dividing by the mean normalized intensity of the protein, achieving a mean value of 1 for each protein across all TMT sets. The proteins detected in at least 30% of samples were included in the statistical analysis, which was performed using the LIMMA package [28]. Protein clustering was performed with the Database for Annotation, Visualization and Integrated Discovery (DAVID) [29]. Ingenuity pathways analysis (IPA, QIAGEN, Hilden, Germany) was used for pathway analysis [30].

### 2.7. Western Blots

The cortical cortex was homogenized with 1× RIPA lysis buffer (Millipore, Burlington, MA, USA; 20-188) and complete protease inhibitor (Millipore, Burlington, MA, USA; 1183617001). The lysate was centrifuged at 15,000× *g*, 4 °C for 10 min; then, we added Laemmli sample buffer (BIO-RAD, Hercules, CA, USA; 1610747); denaturing was performed at 95 °C for 10 min before loading to gel. Proteins were separated on a 4–15% gradient SDS gel, transferred to nitrocellulose paper, and probed overnight at 4 °C with primary antibody as indicated. Antibodies used for Western blotting were rabbit antimouse α-subunit (1:1000; 14676-1-AP; Proteintech, Rosemont, IL, USA); β-subunit (1:1000; 17247-1-AP; Proteintech); c-subunit (1:1000; ab180149; Abcam, Waltham, MA, USA), SerpinA3K (1:1000; 55480-1-AP; Proteintech), HDDC3 (1:1000; 21091-1-AP; Proteintech); and anti-β-tubulin (1:2000; T8328; Sigma, Burbank, CA, USA). Secondary antibodies were antirabbit IgG-HRP (1:1000) and antimouse IgG-HRP (1:1000) from Invitrogen (Carlsbad, CA, USA). Densitometry values from developed and imaged blots were determined using ImageJ 1.54i. For quantification, levels of proteins were normalized to those of β-tubulin and compared using a paired *t*-test.

### 2.8. Electron Microscopy

The animals used for electron microscopy were first perfused transcardially with PBS followed by 4% PFA. The cortical cortex was sliced into 200 μm sections, fixed in 2.5% glutaraldehyde and 2% PFA in 0.1 M cacodylate buffer, post-fixed in 1% osmium tetroxide and 1.5% potassium ferrocyanide in 0.1 M cacodylate buffer, followed by en bloc staining in 2% aqueous uranyl acetate and dehydration in an ethanol series to 100%, and then flat-embedded in Epon. The hardened blocks were sectioned into 60 nm thick slices using a Leica UltraCut UC7. The sections were collected on grids coated with formvar/carbon and contrast stained using 2% uranyl acetate and lead citrate. The grids were imaged on a Tecnai G2 Spirit BioTWIN Transmission EM (ThermoFisher Scientific, Hillsboro, OR, USA) at 80 kV. Images were taken using a NanoSprint15 MKII camera (AMT Imaging, Woburn, MA, USA) using the AMT Capture Engine Software version 7.0.2.5. Over 50 images from two animals in each condition were acquired at a magnification of 4800× and a resolution of at least 400 pixels/µm. To quantify the density of the mitochondrial cristae, images were background-subtracted consistently and converted to binary images in which cristae membranes were black using ImageJ. The mean gray value of the pixels (range 0–255) within the total area of each mitochondrion provided a quantification of cristae density.

## 3. Results

### 3.1. Loss of Slack Channel Activity Alters Expression of Mitochondrial Proteins

We first compared protein expression in the cerebral cortex of wild-type mice to those lacking Slack channel activity. The *Kcnt1* gene in mice comprises 31 exons with two independent promotor regions [31]. In these experiments, we compared the proteomes of wild-type mice with those of mice in which exon 11 of the murine *Kcnt1* gene was deleted [7,26]. This removed the entire six transmembrane-spanning regions that include the K^+^-selective pore domain, which is required for channel activity. While this may not prevent the translation of all possible transcripts from the *Kcnt1* gene, it fully eliminates all ion channel activity. For simplicity, these mice are referred to as *Kcnt1* KO mice.

**Figure 1:**
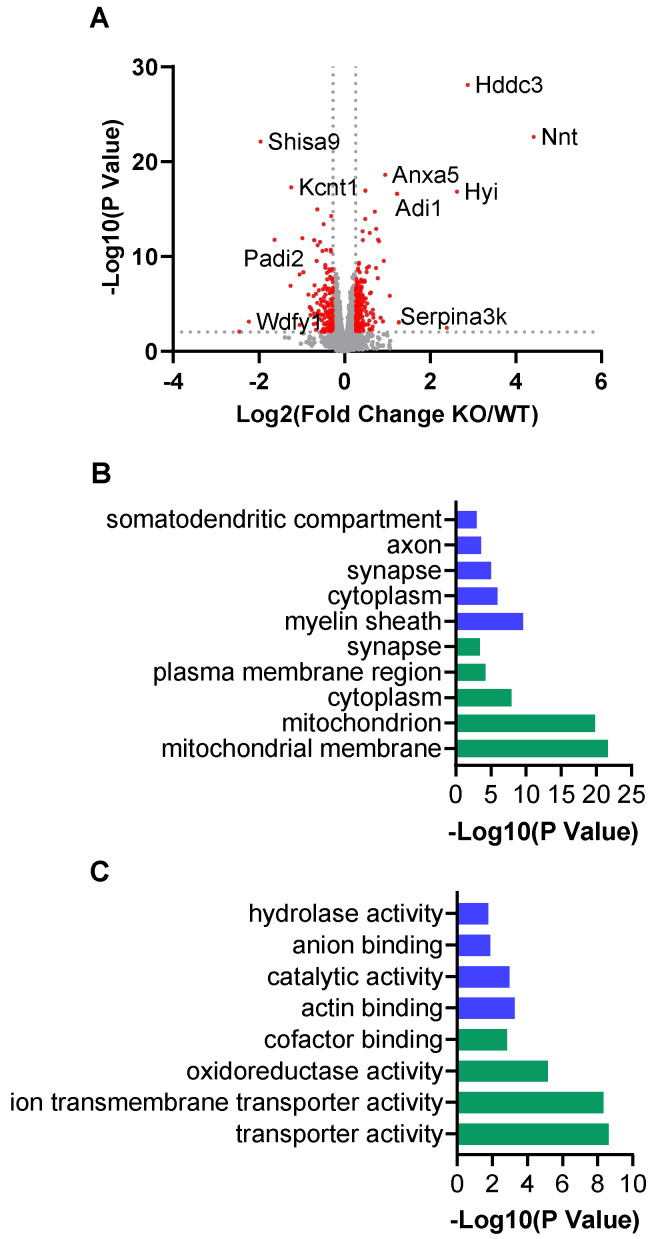
Differential proteomic analysis of *Kcnt1* KO compared to WT mouse cortex. (**A**) Volcano plot showing differentially expressed proteins (DEPs) based on *p* value < 0.01 and |log_2_ fold change| > 0.26. Gene Ontology (GO) enrichment analysis of DEPs in cellular compartment (**B**) and biological process (**C**). Blue and green bars represent down- and upregulated proteins, respectively.

Using tandem mass tag (TMT)-based quantitative proteomics on the cortical extracts from the wild-type and *Kcnt1* KO mice, we quantified 8117 proteins across two sets of TMT-16plex. The median CV of each group of samples was below 7%, and the principal component analysis showed the expected clustering of the same sample groups (Figure S1A,B). Based on a *p* value < 0.01 and a |log_2_ fold change| > 0.26, 361 differentially expressed proteins (DEPs) were identified as altered with loss of Slack activity. Of these, 202 were upregulated, and 159 were downregulated (Figure 1A). Among the severely downregulated proteins was Slack itself, suggesting that loss of the membrane-spanning regions of the channel may still allow translation of some fragments of the proteins but that these are severely attenuated compared to the full channel protein.

Clustering of significantly expressed proteins based on their cellular compartment showed that mitochondrial proteins, and particularly those of the inner mitochondrial membrane, were highly enriched among the proteins that had an increased abundance. In contrast, proteins of the myelin sheath, axon, and somatodendritic compartment were over-represented in proteins with decreased expression. Both the up- and downregulated protein groups were enriched for proteins located at synapses (Figure 1B and Table S1), and it was interesting to find that some upregulated proteins were involved in neuroactive ligand–receptor interaction such as Pdyn, Gabra2, and Npy1r. Based on their biological functions, the upregulated proteins were involved in ATP metabolic processes, as well as mitochondrial transmembrane transport and organization (Figure 1C). In contrast, the downregulated proteins were related to myelination, neurogenesis, and axonal development (Figure 1C).

**Figure 2:**
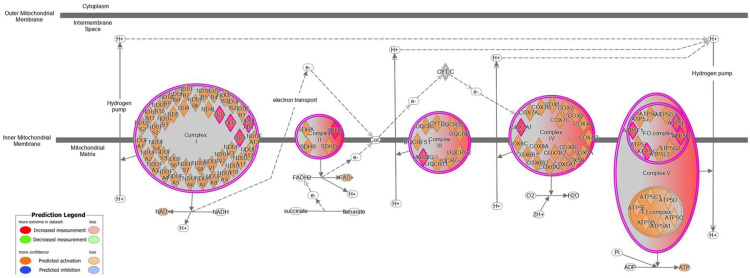
Schematic of proteins of the oxidative phosphorylation pathway in the inner mitochondrial membrane. Proteins that were significantly upregulated in the cortex of *Kcnt1* KO mice are colored in red.

As an alternative method of characterizing the biological pathways affected by *Kcnt1* KO, we carried out ingenuity pathway analysis (IPA) on the differentially expressed proteins. The top enriched pathways included mitochondrial dysfunction and oxidative phosphorylation, metabolic pathways, and immune response (Figure S2). Fourteen of the upregulated proteins participate in mitochondrial oxidative phosphorylation including Atp5mf, Atp5mg, Atp5pb, Atp5pd, Cox15, Cox6c, Cox7a1, Dmac2l, Mtnd1, Mtnd2, Mtnd3, Ndufab1, Sdhc, and Uqcrq (Figure 2).

**Figure 3:**
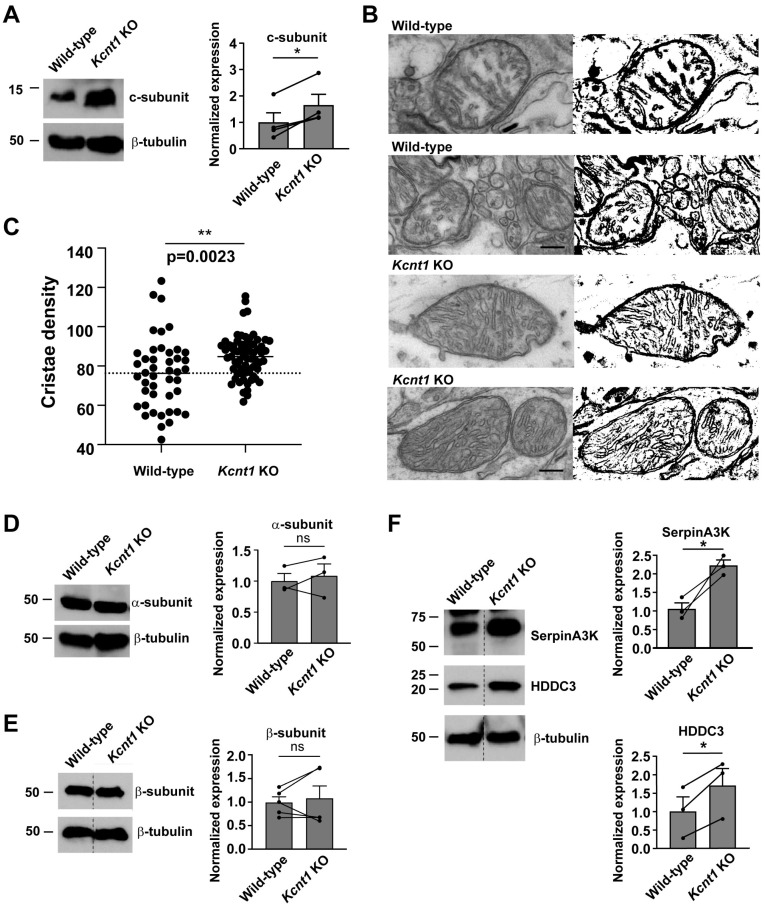
Changes in protein expression and mitochondrial structure in *Kcnt1* KO cortical cortex. (**A**) Immunoblotting of c-subunit of ATP synthase in cortex. Right panel shows quantification (n = 4). (**B**) Transmission electron microscopy images of mitochondria in pyramidal neurons of wild-type and *Kcnt1* KO cortex (left row). The images were converted to binary images (right row) for quantification with ImageJ. (**C**) Quantification of density of cristae from binary images (see Section 2). Dots represent crista density in individual mitochondria (unpaired *t*-test). (**D**–**F**) Immunoblots of the α-subunit (**D**) and β-subunit (**E**) of the mitochondrial ATP synthase, as well as of SerpinA3K and HDDC3 (**F**) in wild-type and *Kcnt1* KO cortices. Quantification is shown in right panels right (n = 3–5, * *p* < 0.05, ** *p* < 0.01 paired *t*-test; ns, not significant). Original Western blot images are provided in the Supplementary Materials.

To examine the impact of loss of Slack activity on mitochondrial pathways and structure directly, we next isolated cortical tissue from wild-type and *Kcnt1* KO mice and carried out Western blotting for Atp5g, the c-subunit of mitochondrial ATP synthase. In addition to its role in ATP production, this subunit regulates the formation of cristae by dimerizing at the apex of cristae membranes [32]. The levels of the c-subunit were found to be significantly increased in the cortex of *Kcnt1* KO animals (Figure 3A). Finally, we carried out electron microscopy to examine the structure of the mitochondria in the fixed tissue from the cerebral cortices of the wild-type and *Kcnt1* KO mice. The effect of the loss of *Kcnt1* channel activity was clearly apparent by visual inspection (Figure 3B). Specifically, the mitochondria in the neurons from *Kcnt1* KO mice had a greatly increased number of cristae per unit area, with a concomitant decrease in the volume of the mitochondrial matrix. This was quantified by generating binary black-and-white images of the mitochondria in which cristae membranes appear black, while matrix and intermembrane spaces appear white (Figure 3B). The ratio of black to white pixels within the internal area of the mitochondrial provided a quantification of the density of cristae. This was found to be very significantly increased in *Kcnt1* KO neurons compared with those in the wild type (Figure 3C).

The mitochondrial ATP synthase is composed of two multisubunit complexes: the Fo component that spans the inner mitochondrial membrane and the soluble F1 component that resides on the matrix side of this membrane. Interestingly, most of the ATP synthase subunits that were found to be markedly increased in the proteome of the *Kcnt1* KO mice were subunits of the Fo membrane complex, while only little or no change was found for some of the components of F1, such as the α-subunit (Atp5f1a) and the β-subunit (Atp5f1b). We therefore also carried out Western blotting to determine whether the levels of the α- or β-subunits were altered in *Kcnt1* KO animals. We found no significant differences in the levels of these subunits between the wild-type and knockout animals (Figure 3D,E). Although we cannot eliminate the possibility that small changes may become evident with further work, our findings suggest that the loss of Slack channel activity may alter the stoichiometry of the different components of ATP synthase.

Among the other proteins significantly increased in the proteome of the cortex of *Kcnt1* KO mice was SerpinA3K, a serine protease inhibitor. Consistent with the observed increases in inner mitochondrial membrane proteins, SerpinA3K has been found to protect cells against oxidative stress [33,34], a likely consequence of increased mitochondrial respiration. The increase in SerpinA3K was confirmed by Western blotting of extracts of wild-type and *Kcnt1* KO cortices (Figure 3F). Another protein significantly enriched in the proteome of the *Kcnt1* KO cortex and confirmed by Western blotting was Hddc3 (Figure 3F). This is a cytosolic NADPH phosphatase termed Mesh1, an enzyme that also regulates cell responses to oxidative stress [35,36,37].

### 3.2. Antisense Oligonucleotides Against Kcnt1 in Adult Animals Do Not Produce the Same Changes as Loss of Slack Channel Activity During Development

The complete loss of Slack channel activity during development, as in the case of the *Kcnt1* KO animals, might be expected to have different effects on the proteome compared to the suppression of *Kcnt1* expression in older wild-type animals. For example, compensatory mechanisms for the loss of channel activity during early development may be absent from later developmental stages. Using TMT-based quantitative proteomics, we therefore further systematically explored the effects of a *Kcnt1* ASO on the proteome of the wild-type mouse cortex.

**Figure 4:**
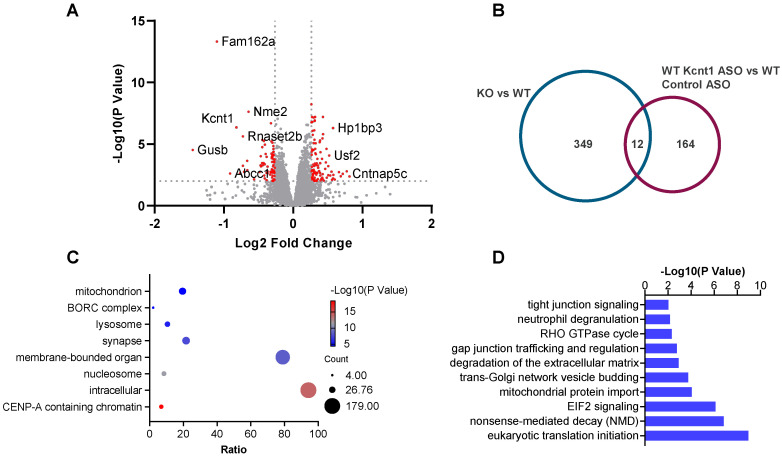
(**A**) Volcano plot of protein abundance change in WT mouse cortex with *Kcnt1* ASO treatment compared to control ASO (*p* value < 0.01 and |log_2_ fold change| > 0.26). (**B**) Venn diagram of DEPs from WT mice with *Kcnt1* ASO treatment and *Kcnt1* KO mice. Functional (**C**) and pathway (**D**) analyses of DEPs found in WT mice treated with *Kcnt1* ASO compared to control ASO.

In the experiments with *Kcnt1* ASO, a total of 8236 proteins were quantified across two sets of TMT-18plex. Based on a *p* value < 0.01 and a|log_2_ fold change| > 0.26, 176 DEPs were identified in the wild-type mouse brains that received *Kcnt1* ASO treatment (Figure 4A). The ASO significantly decreased *Kcnt1* protein levels in the wild-type mice (Figure S3A). Functional annotation revealed that DEPs with *Kcnt1* ASO treatment were located in various cellular compartments including the cytoplasm, mitochondrion, lysosome, and synapse and were involved in processes associated with cytoplasmic translation, metabolism, and oxidation reduction (Figure 4C). Pathway analysis indicated that the DEPs from the wild-type mice were enriched in eukaryotic translation initiation, mitochondrial protein import, and trans-Golgi network vesicle budding (Figure 4D).

To further investigate the effects of *Kcnt1* ASO, we compared the proteome changes between KO mice and wild-type mice with *Knct1* ASO treatment. Only 12 DEPs were found in both the *Kcnt1* KO mice and wild-type mice with *Kcnt1* ASO treatment (Figure 4B). The following proteins showed the same direction of change: Kcnt1, Cldn11, Bcas1, Ermn, Aspa, Yjefn3 (decreased abundance), and Pdyn (increased abundance). Conversely, Nme1, Fhit, Cck, Hist1h4a, and Fam162a exhibited the opposite changes. The fold change of *Kcnt1* in KO mice (FC = 0.42) was smaller than in wild-type mice with ASO treatment (FC = 0.57). The mitochondrial membrane proteins that were markedly altered in the *Kcnt1* KO mice did not show significant abundance changes in the wild-type mice with *Kcnt1* ASO treatment, except for Atp5pb (FC = 0.90, *p* = 1.65 × 10^−3^), Atp5mf (FC = 0.90, *p* = 2.34 × 10^−3^), and Cox6c (FC = 0.84, *p* = 6.30 × 10^−3^), which showed an opposite direction of change compared to that in *Kcnt1* KO mice. Meanwhile, four mitochondrial inner membrane proteins, including Mthfd2l, Cox7b, Uqcrc1, and Timm8a1, were downregulated in the ASO-treated wild-type mice, while their abundance remained unchanged in the *Kcnt1* KO mice.

### 3.3. A Gain-of-Function Kcnt1 Mutation Alters the Molecular Profile of Cortical Neurons

In mice, loss of *Kcnt1* produces learning deficits but does not result in seizures [7,13]. Gain-of-function mutations of *KCNT1* in humans and mice disrupt learning but also produce increased cortical excitability and increased sensitivity to seizure-provoking stimuli [13,38,39]. As one example, *Kcnt1*^R455H/+^ mice experience spontaneous seizures and persistent interictal spikes [13], as is also the case for humans that are heterologous for the corresponding mutation *KCNT1-R474H* [11]. At the cellular level, this mutation has been found to increase the intrinsic excitability of cortical pyramidal neurons but to suppress that of inhibitory interneurons [14]. We therefore compared the cortical proteome of wild-type mice with that of *Kcnt1*^R455H/+^ mice and tested the effect of *Kcnt1* ASO on these mutant mice.

**Figure 5:**
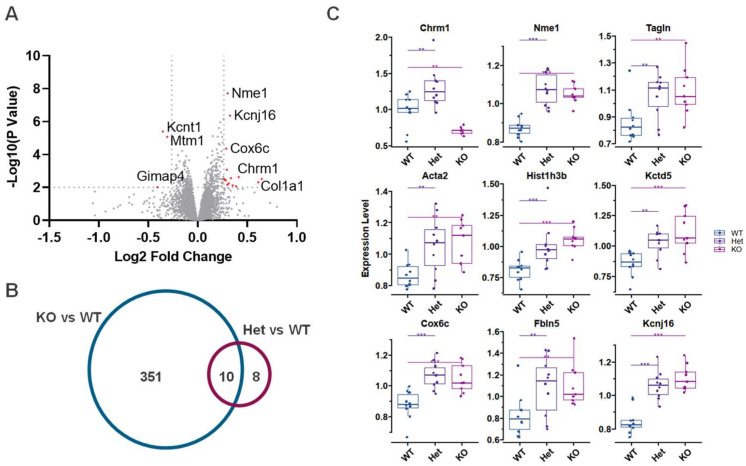
(**A**) Volcano plot of protein abundance change between *Kcnt1* heterozygous mutation (*Kcnt1^R455H/+^*, Het) and WT mouse cortex. (**B**) Venn diagram of DEPs from KO and *Kcnt1^R455H/^*^+^ mice relative to WT mice. (**C**) Expression of the overlapped DEPs in *Kcnt1^R455H/+^* (Het) and KO mice. Unpaired *t*-test: ** *p* < 0.01, *** *p* < 0.001.

Using the criteria of a *p* value < 0.01 and a |log_2_ fold change| > 0.26, we found only 18 DEPs between the *Kcnt1^R455H/+^* mice and wild-type mice (Figure 5A and Figure S4), suggesting that this *Kcnt1* mutation produces relatively small changes to the brain proteomes of adult mice. Ten proteins were found to be differentially expressed in both *Kcnt1* KO and *Kcnt1*^R455H/+^ animals, with the majority being upregulated in both the knockout and the *Kcnt1*^R455H/+^ mutant mice (Figure 5B). These included the K^+^ channel Kcnj16 (Kir5.1), the K^+^ channel tetramerization domain-containing 5 protein Kctd5, as well as the nucleoside diphosphate kinase-1 (Nme1) (Figure 5C). Levels of the *Kcnt1* protein were found to be decreased in the *Kcnt1^R455H/+^* animals, as were the levels of the dual-specificity phosphatase Mtm1. The changes in Nme1 and Mtm1 suggest that the mutant channels may produce changes in cellular metabolism. Proteins that have been found to interact with *Kcnt1* directly [22,24,40,41], including Fmr1, Eif4e, Phactr1, and Cyfip1, showed no changes in expression in either the *Kcnt1^R455H/+^* or KO mice (Figure S5). Most of the mitochondrial membrane proteins that were markedly altered in *Kcnt1* KO mice did not show significant abundance changes in *Kcnt1^R455H/+^* mice, except for Cox6c (FC = 1.22, *p* = 4.43 × 10^−5^), Atp5mf (FC = 1.14, *p* = 3. × 10^−4^), Atp5pb (FC = 1.15, *p* = 8.82 × 10^−4^), and Uqcrq (FC = 1.15, *p* = 8.48 × 10^−5^), which displayed the same direction of abundance change as in the *Kcnt1* KO mice. Interestingly, only one protein, muscarinic acetylcholine receptor M1 (Chrm1), showed an opposite direction of change, being significantly increased in *Kcnt1^R455H/+^* mice and downregulated with *Kcnt1* KO. These data indicate that the changes produced by the gain-of-function mutation are not simply the inverse of the changes observed with the loss of function.

Because of the limited changes that were detected between the wild-type and *Kcnt1*^R455H/+^ mice using the criterion of |log_2_ fold change| > 0.26, we repeated the analysis using only the criterion of a *p* value < 0.01 to capture a broader spectrum of the potential effects from the *Kcnt1* mutation. Using this criterion, 274 proteins were found to be dysregulated in the *Kcnt1^R455H/+^* mice. Pathway analysis indicated that several pathways were over-represented in the *Kcnt1^R455H/+^* mice, including oxidative phosphorylation, mitochondrial protein import, and ion channel transport (Figure S6). Interestingly, multiple mitochondrial inner membrane proteins, such as Atp5pb, Cox7a2, and Ndufa3, underwent a significant increase in abundance, although the fold change was small (FC < 1.2). These results are further discussed in the next section on the use of ASO to modify protein expression in the mutant animals.

### 3.4. ASO Suppression of Kcnt1 Expression Partially Rescues the Proteomic Phenotype of Mutant Mice

**Figure 6:**
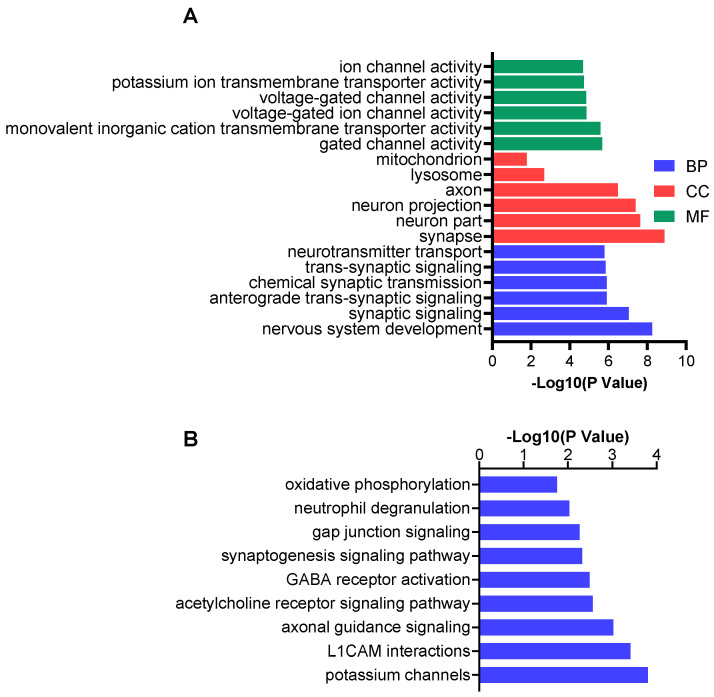
Functional (**A**) and pathway (**B**) analyses of DEPs found in mutant mice treated with *Kcnt1* ASO compared to control ASO.

Using the same criteria of a *p* value < 0.01 and a |log_2_ fold change| > 0.26, we found 123 DEPs in the brains of the *Kcnt1^R455H/+^* mice treated with *Kcnt1* ASO compared to those treated with the control ASO. These DEPs are involved in various synaptic processes including synaptic signaling, neurotransmitter transport, and displayed ion channel activity and potassium ion transmembrane transporter activity (Figure 6A). In terms of cellular compartments, the synapse, axon, and neuron projection were over-represented (Figure 6A). The top enriched pathways for the DEPs in the *Kcnt1^R455H/+^* ASO mice included potassium channels, L1CAM interactions, axonal guidance signaling, and synaptogenesis signaling (Figure 6B). Four inner mitochondrial membrane proteins, including Ndufv3 (FC = 0.78, *p*= 9.74 × 10^−3^), Ghitm (FC = 0.82, *p*= 1.82 × 10^−3^), Cox7b (FC = 0.80, *p*= 7.65 × 10^−4^), and Uqcrc1 (FC = 0.80, *p*= 3.36 × 10^−5^), were downregulated in the mutant mice with ASO treatment. Functional annotation revealed that while cellular compartments and pathways affected by *Kcnt1* ASO in the wild-type and *Kcnt1^R455H/+^* animals were different, there were also some commonalities. More than 30 of these DEPs were altered by ASO treatment in both the wild-type and *Kcnt1^R455H/+^* animals (Figure S3B). Seven of these, including Dtnb, Dnm2, Nrgn, Stx3, Asic2, Ntm, and Gpc6, were annotated as synaptic proteins, and all displayed the same direction of change in response to *Kcnt1* ASO treatment compared to the corresponding control groups (Figure S3C). The inner mitochondrial membrane proteins Cox7b and Uqcrc1were downregulated in both the WT and mutant mice with ASO treatment (Figure S3C). Pathway analysis also identified neutrophil degranulation as enriched in both strains.

As described in the previous section, only a small set of proteins (18) were found to be altered in their expression levels in the cortices of *Kcnt1*^R455H/+^ mice compared to the wild-type mice using the criteria of a *p* value < 0.01 and a |log_2_ fold change| > 0.26. We next investigated whether the *Kcnt1* ASO could rescue the proteomic phenotype of the mutant mice by comparing the expression levels of proteins in the wild-type animals treated with the control ASO with those of the mutant mice treated with either the control ASO or the *Kcnt1* ASO. Using the same criteria, we found only three DEPs (Agt, Cd34, and Nap1l5) whose levels differed in wild-type mice compared to those in the mutant animals treated with the control ASO and were altered by treatment with the *Kcnt1* ASO. Notably, *Kcnt1* ASO treatment restored the levels of Agt and Nap1l5 to those in the wild type (Figure S7).

**Figure 7:**
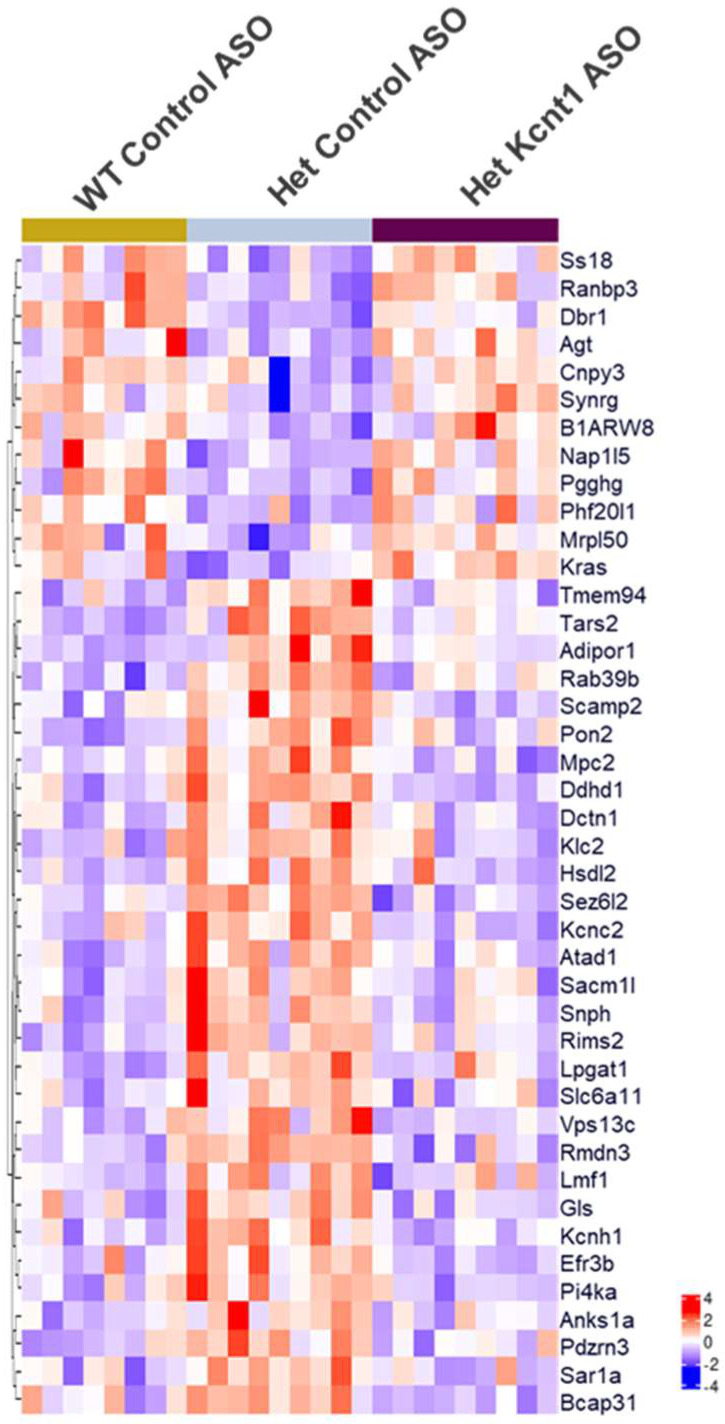
Heatmap of proteins dysregulated by mutation (Het, *Kcnt1^R455H/+^*) and then rescued by *Kcnt1* ASO treatment.

Because the changes in the abundance of biologically relevant proteins in response to either the *Kcnt1*^R455H/+^ mutation or to ASO treatment may be small, we further analyzed the effects of the *Kcnt1* ASO using only the criterion of a *p* value < 0.01. This would be expected to capture a broader spectrum of the potential effects from both the *Kcnt1* mutation and the *Kcnt1* ASO treatment and identify additional potential targets for future research. With this criterion, 42 proteins were found to be dysregulated in the *Kcnt1^R455H/+^* mice, and their expression levels restored to wild-type levels with *Kcnt1* ASO treatment (Figure 7). Proteins related to synapses (Atad1, Pdzrn3, Sacm1l, Gls, Kcnc2, Kcnh1, Rims2, Slc6a11, Snph) and proteins with ion transmembrane transporter activity (Mpc2, Kcnc2, Kcnh1, Slc6a11) were found to be increased in the mutant mouse brain, but their expressions were restored with *Kcnt1* ASO treatment. It was also found that the *Kcnt1* ASO was able to attenuate the expression of several mitochondrial proteins including Atad1, Gls, Mpc2, Rmdn3, and Tars2, of which the mitochondrial membrane proteins Atad1, Mpc2, and Rmdn3 were significantly increased in the mutant mice.

## 4. Discussion

Changes in either the levels or activity of the Slack potassium channel have major effects on the ability of the nervous system to carry out essential brain functions, including learning and memory. In mice, the loss of Slack channel function early in development causes complete loss of hippocampal LTP and LTD, two forms of synaptic plasticity that are generally thought to underlie many forms of learning [6]. At least in part, this is associated with reduced levels and signaling of the NMDA receptor as well as reduced dephosphorylation of the AMPA receptor subunit GluA1 in response to stimulation. During development, however, homeostatic mechanisms restore some forms of synaptic plasticity. Thus, in adults such as those used in the present study, LTP and some but not all forms of LTD can be detected. Nevertheless, abnormalities persist in the adult animals, who show severe cognitive inflexibility in behavioral tests [7].

Our present proteomic studies, coupled with Western blots and electron microscopy, revealed that a major consequence of the loss of Slack channel activity is an increase in proteins required for oxidative phosphorylation within the inner mitochondrial membrane. This is consistent with the increase in mitochondrial cristae, which increase the surface area available for mitochondrial respiration. Moreover, dimers of the mitochondrial ATPase promote cristae formation by allowing the tight folding of the inner membrane at the tips of the cristate [32]. Emerging evidence suggests that changes in mitochondrial metabolism play an essential role in synaptic plasticity [42,43]. Moreover, recent super-resolution techniques have shown that cristae membranes undergo dynamic remodeling in living cells [44,45,46]. This occurs on a time scale of seconds and reflects changes in oxidative phosphorylation and ATP–ADP exchange across the inner membrane. Our data indicate that the mitochondria in the cerebral cortex of wild-type animals have a much broader range of cristae densities than those of Slack KO animals, which have a much more uniform distribution, but with an overall higher density. One possible explanation for this is that the loss of Slack channels disrupts the rapid adaptation of mitochondria to metabolic demands, but further work will be required to test this. The overall higher expression of proteins involved in oxidative phosphorylation in the Slack KO animals is also consistent with the upregulation of proteins such as SerpinA3K and Hddc3 that control cell responses to oxidative stress.

The changes that were detected between the proteomic profiles of *Kcnt1^R455H/+^* and wild-type mice were generally much smaller than those produced by the elimination of Slack channel function. As a result, verification of such changes by Western blotting and morphological analyses may be more challenging. Moreover, it is likely that other significant biological pathways that are altered by such gain of function could be difficult to detect in proteomic studies of whole cortical tissue. For example, the levels of the sodium channel subunit Na_V_1.6 are upregulated in the cerebral cortex of 2-month-old *Kcnt1^R455H/+^* mice, and Na_V_1.1 and Na_V_1.2 levels are increased in neurons cultured from these animals at much earlier stages of development [14]. These changes were also evident in patch clamp recordings of Na^+^ currents and in the length of the axon initial segments to which Na_V_1.6 is localized. The relative low abundance of such proteins, coupled with their specific localization to discrete cellular compartments, may impede their detection in classic proteomic screens.

ASO targeting of RNA transcripts is an innovative therapeutic modality and has already been demonstrated to be an effective treatment for multiple diseases [47,48]. Growing evidence also supports the development of ASO-based strategies as viable therapies for epilepsy [49,50]. An ASO targeting *Kcnt1* was proven to reduce seizures and increase life span in an animal model [25]. Here, quantitative proteomics was used to systematically investigate the molecular profile changes in wild-type and *Kcnt1^R455H/+^* mutant mice following *Kcnt1* ASO treatment. The proteomics results revealed alterations in the expressions of multiple synaptic proteins including Dtnb, Dnm2, Nrgn, Stx3, Asic2, Ntm, and Gpc6 in both wild-type and *Kcnt1^R455H/+^* mice following ASO treatment. These proteins are crucial for synaptic function, including neurotransmitter release, synaptic vesicle cycling, and signal transduction. The same direction of abundance change in these proteins across the wild-type and mutant mice suggests a robust effect of reducing *Kcnt1* expression on synaptic integrity and function. Interestingly, the pathway of neutrophil degranulation that is closely correlated with inflammatory responses was enriched in both the wild-type and mutant mice following ASO treatment. A previous study indicated that the administration of certain ASOs might trigger the innate immune system in the mouse brain [51]. Different proteomic changes were also observed in wild-type and *Kcnt1^R455H/+^* mutant mice with the reduced expression of *Kcnt1* following ASO treatment. In the wild-type mice, ASO treatment disrupted normal Slack function, leading to compensatory changes such as translation initiation, mitochondrial protein import, and trans-Golgi network vesicle budding, likely to maintain cellular homeostasis. In contrast, the ASO treatment of mutant mice corrected neuronal excitability and synaptic function by restoring proteomic profiles related to synaptic signaling, neurotransmitter transport, ion channel activity, synaptogenesis, and neuronal projections.

## 5. Conclusions

Our findings indicate that complete loss of Slack channel activity during development suppresses the expression of multiple proteins that localize to axons, dendrites and myelin sheath, but increases levels of inner mitochondrial membrane proteins, leading to increased packing of mitochondrial cristae membranes. The mechanisms by which these changes occur and their consequences for neuronal metabolism have yet to be determined. We also found that a smaller but distinct set of changes, including some in mitochondrial proteins, is produced by a clinically relevant gain-of-function channel mutation, as well as by treating wild-type and mutant animals with an ASO targeted to the channel. Reducing *KCNT1* expression through an ASO is a promising therapeutic strategy for epilepsy caused by gain-of-function *KCNT1* mutations. Our proteomics profiling revealed that the relatively small changes in proteins involved in synaptic signaling, ion transport, and oxidative stress response could be reversed by the reduction of *Kcnt1* in mutant mice. The restoration of these proteins suggests that ASO treatment effectively counteracts the proteomic disruptions caused by the *Kcnt1*^R455H/+^ mutation, thereby restoring cellular homeostasis and neuronal functions. This is in line with the observed phenotype rescue including seizure reduction and life extension observed in mouse models with *Kcnt1* ASO treatment [25]. These results indicate that the disease trajectories of proteins and associated pathways that are dependent on *Kcnt1* can be reversed, supporting the development of such agents for therapeutic approaches to the conditions caused by gain-of-function *KCNT1* mutations in humans.

## Data Availability

The mass spectrometry data were deposited to the public MassIVE database. The identifier is MSV000095831.

## Supplementary Materials

The following supporting information can be downloaded at: https://www.mdpi.com/article/10.3390/biom14111397/s1, Figure S1: (A) Coefficient of variation (CV) distribution of all quantified proteins by TMT-based quantitative proteomics. (B) Principal component analysis (PCA) of the proteins in each group. Figure S2: Top enriched pathways of differentially expressed proteins in KO compared to WT mice. The pathway result was generated through ingenuity pathway analysis (IPA, Qiagen). Figure S3: (A) *Kcnt1* expression in WT (control ASO and Kcnt1 ASO) and *Kcnt1^R455H/+^* (Het, control ASO and Kcnt1 ASO) mice. (B) Venn diagram of DEPs from WT and *Kcnt1^R455H/+^* mice with *Kcnt1* ASO treatment. (C) DEPs annotated as synapse and mitochondrial inner membrane proteins and found in both WT and *Kcnt1^R455H/+^* mice with Kcnt1 ASO treatment. Figure S4: The string network of all the differentially expressed proteins in the *Kcnt1^R455H/+^* mice. Figure S5: Proteins interacting with Kcnt1 showed no changes in *Kcnt1^R455H/+^* (Het) and KO mice compared to WT mice. Figure S6: Top enriched pathways of proteins with significant abundance changes (*p* value < 0.01) in mutant mice compared to WT mice. The pathway result was generated through ingenuity pathway analysis (IPA, Qiagen). Figure S7: DEPs found in both mutant and *Kcnt1^R455H/+^* mice with Kcnt1 ASO treatment (*p* value < 0.01 and |log_2_ fold change| > 0.26). WT.Ctl: WT mice with control ASO treatment; Het.Ctl: mutant mice with control ASO treatment; Het.KCNT1: mutant mice with Kcnt1 ASO treatment. Table S1: Up-and down-regulated genes related to GO terms of synapse and cytoplasm in Kcnt1 KO mice.
