# Supplementary material for: Molecular Profiling of Mouse Models of Loss or Gain of Function of the KCNT1 (Slack) Potassium Channel and Antisense Oligonucleotide Treatment
Source: Biomolecules. 2024 Nov 2;14(11):1397. doi: 10.3390/biom14111397 (PMC11591899; doi:10.3390/biom14111397)
Supplement: Supplementary file 1 [file biomolecules-14-01397-s001.zip › biomolecules-3233554-original-images.pdf]

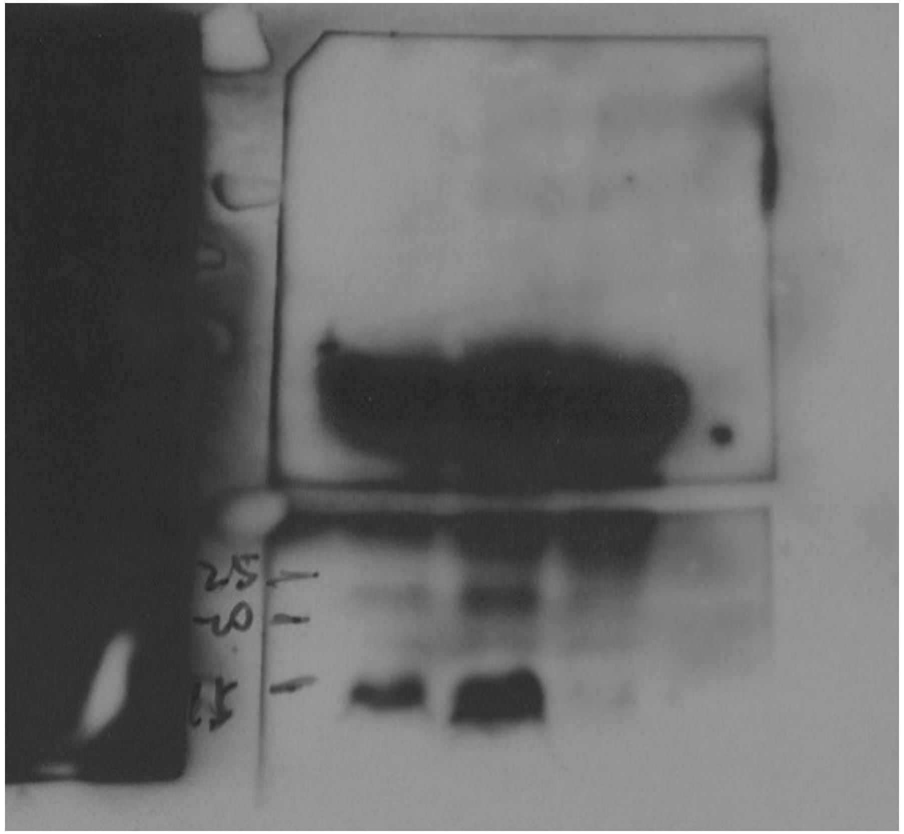

c-subunit

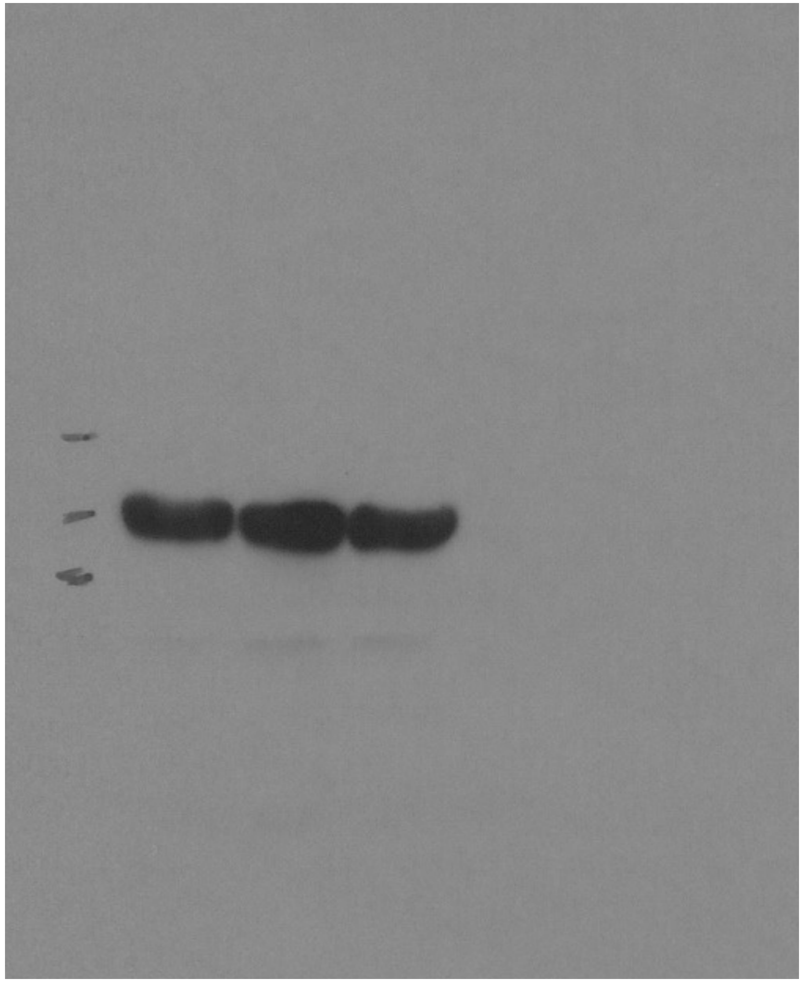

β-tubulin

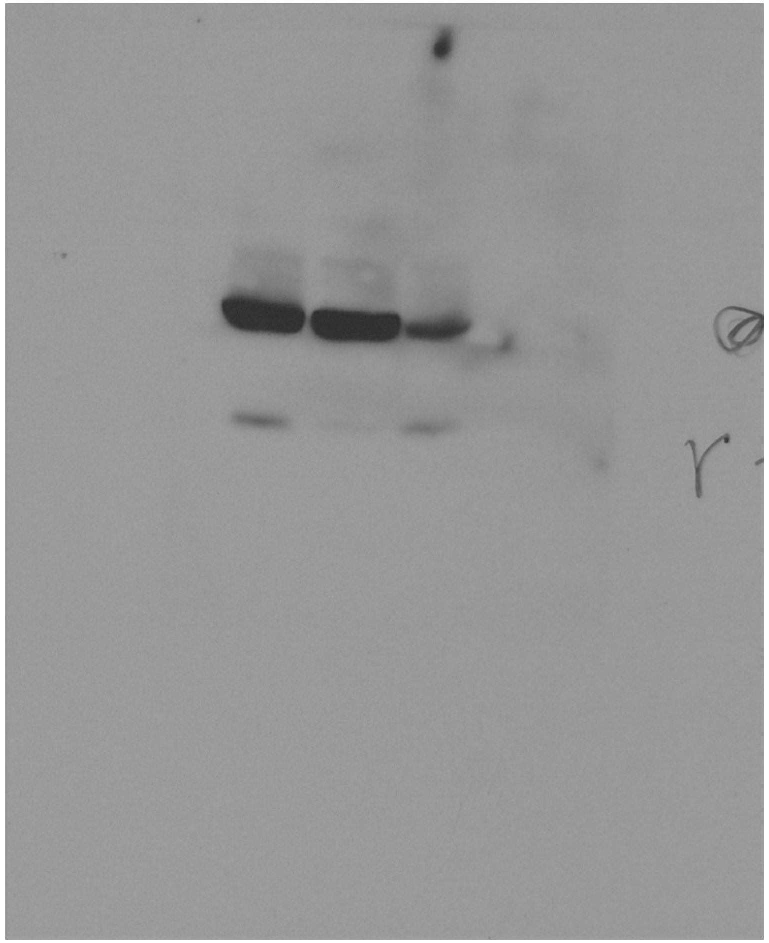

α-subunit

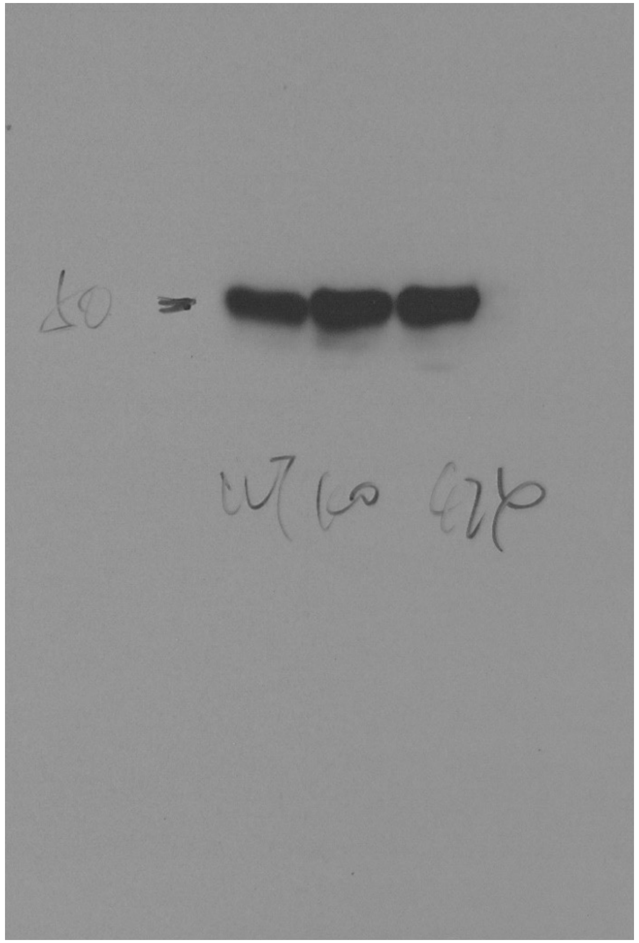

β-tubulin

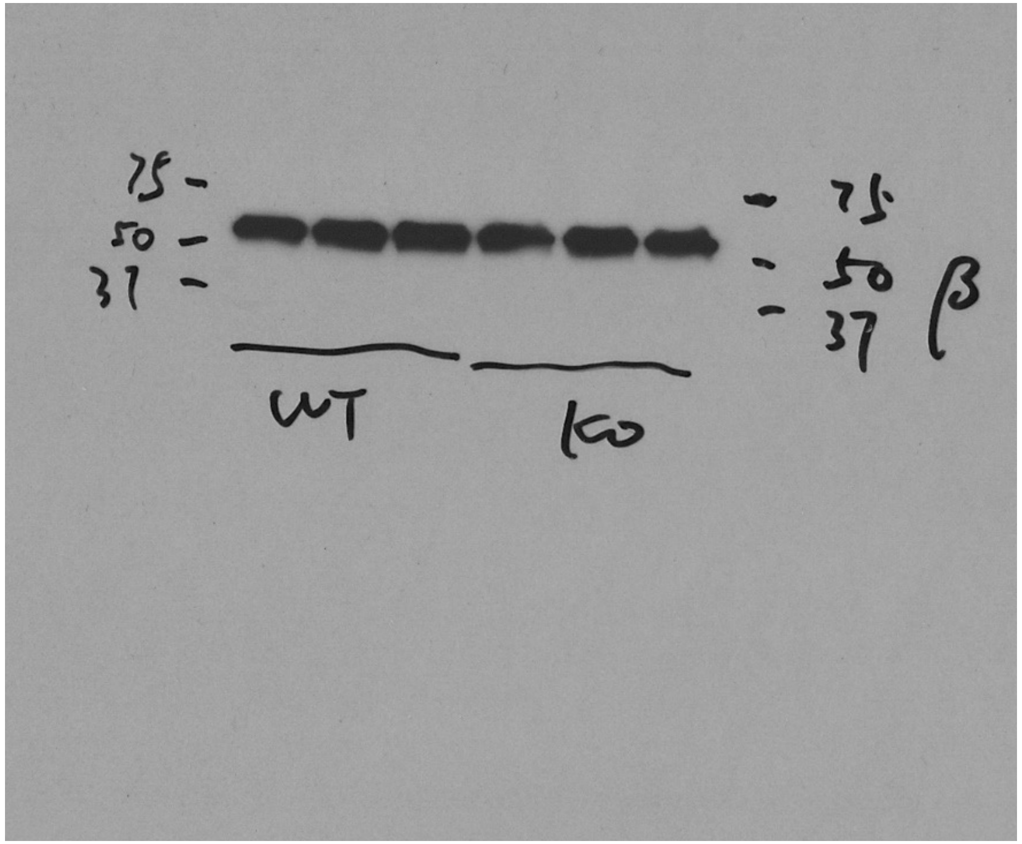

β-subunit

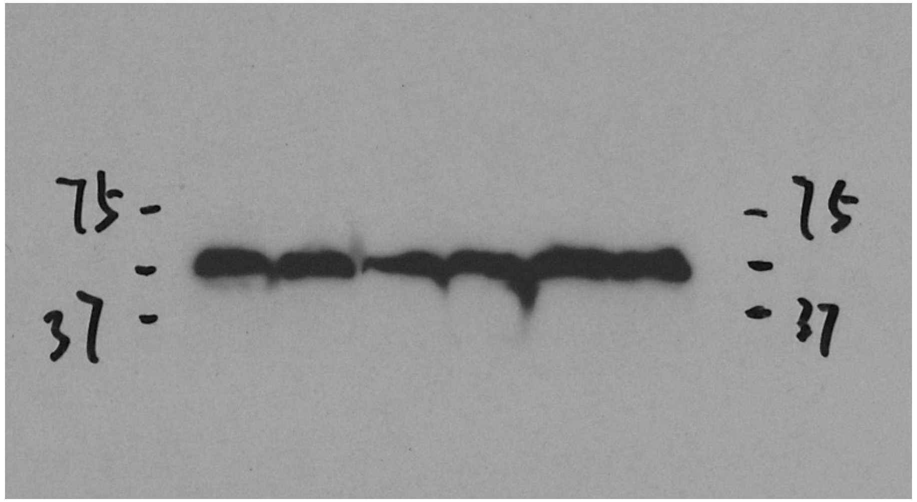

β-tubulin

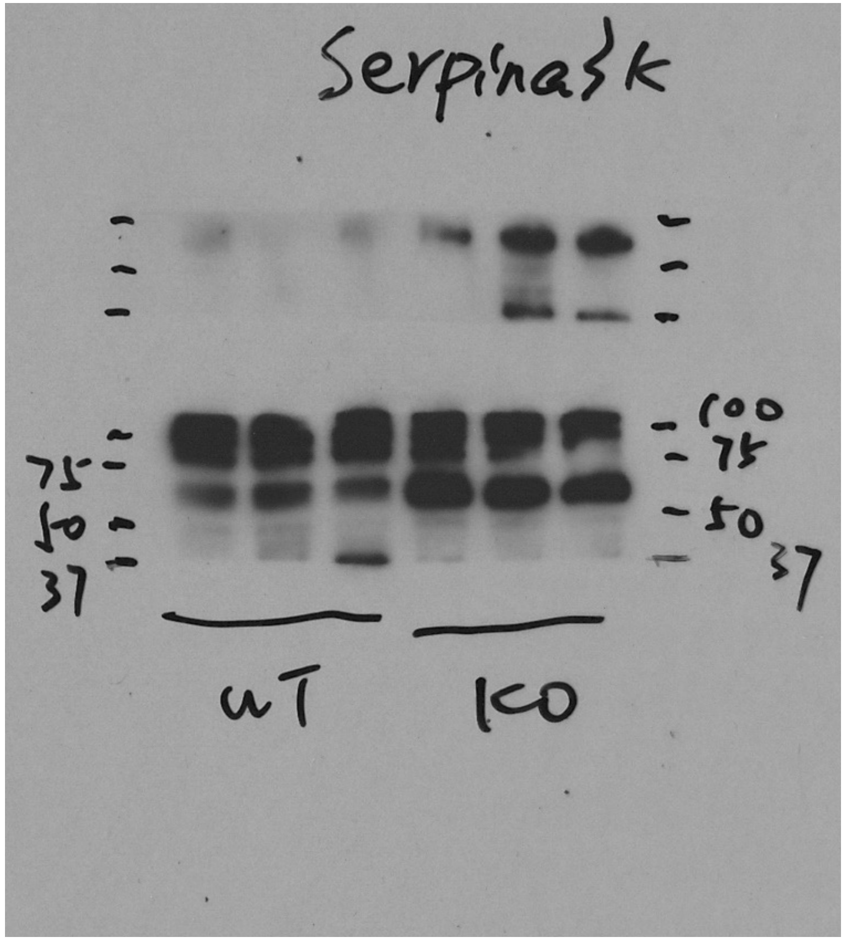

SerpinA3K (47kD)

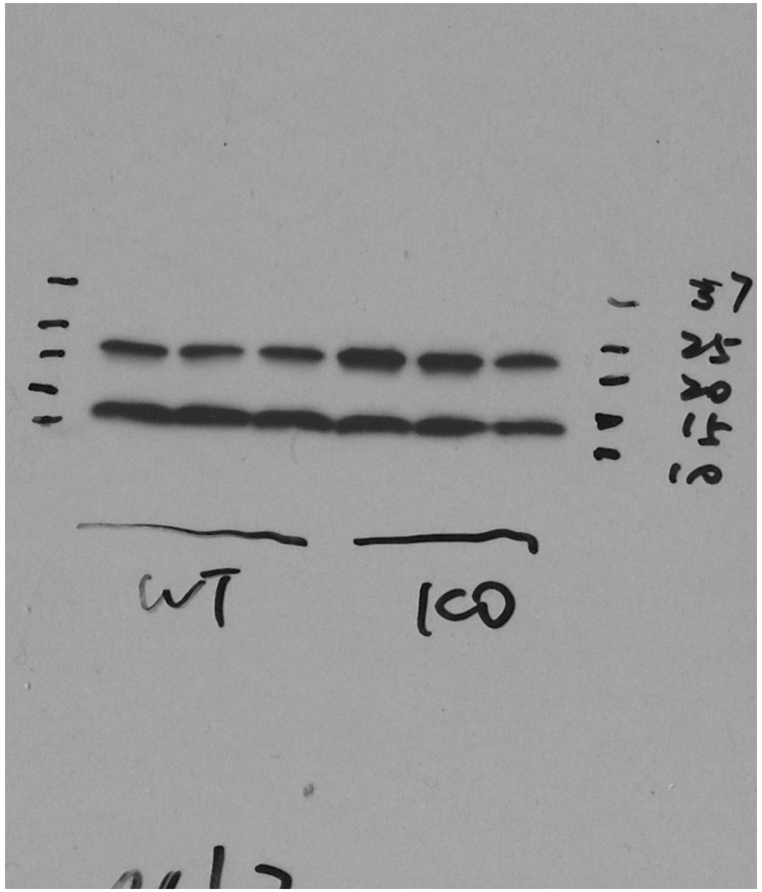

HDDC3 (20 kD)

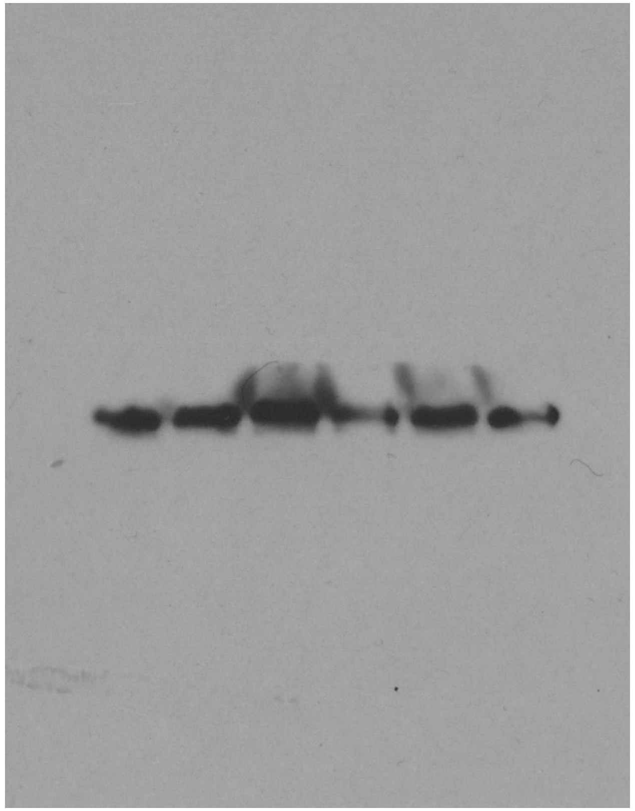

β-Tubulin
