# Supplementary material for: Molecular Profiling of Mouse Models of Loss or Gain of Function of the KCNT1 (Slack) Potassium Channel and Antisense Oligonucleotide Treatment
Source: Biomolecules. 2024 Nov 2;14(11):1397. doi: 10.3390/biom14111397 (PMC11591899; doi:10.3390/biom14111397)
Supplement: Supplementary file 1 [file biomolecules-14-01397-s001.zip › biomolecules-3233554-supplementary.pdf]

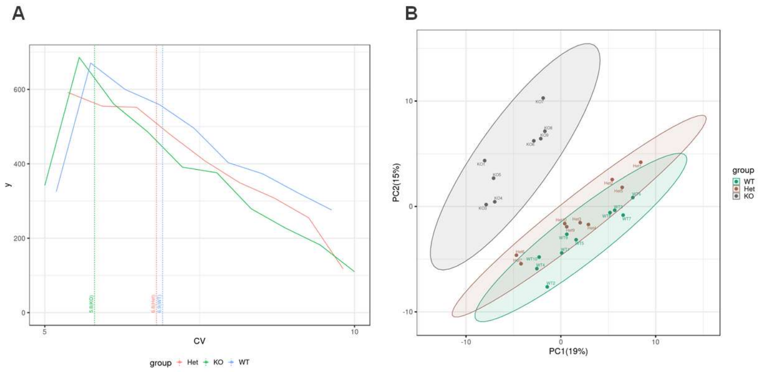

Figure S1. (A) Coefficient of variation (CV) distribution of all quantified proteins by TMT-based quantitative proteomics. (B) Principal component analysis (PCA) of the proteins in each group. Het: *Kcnt1*$^{R455H/+}$.

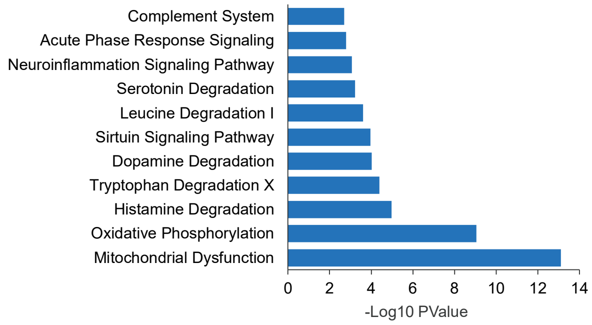

Figure S2. Top enriched pathways of differentially expressed proteins in *Kcnt1* KO mice compared to WT mice. The pathway result was generated through Ingenuity Pathway Analysis (IPA, Qiagen).

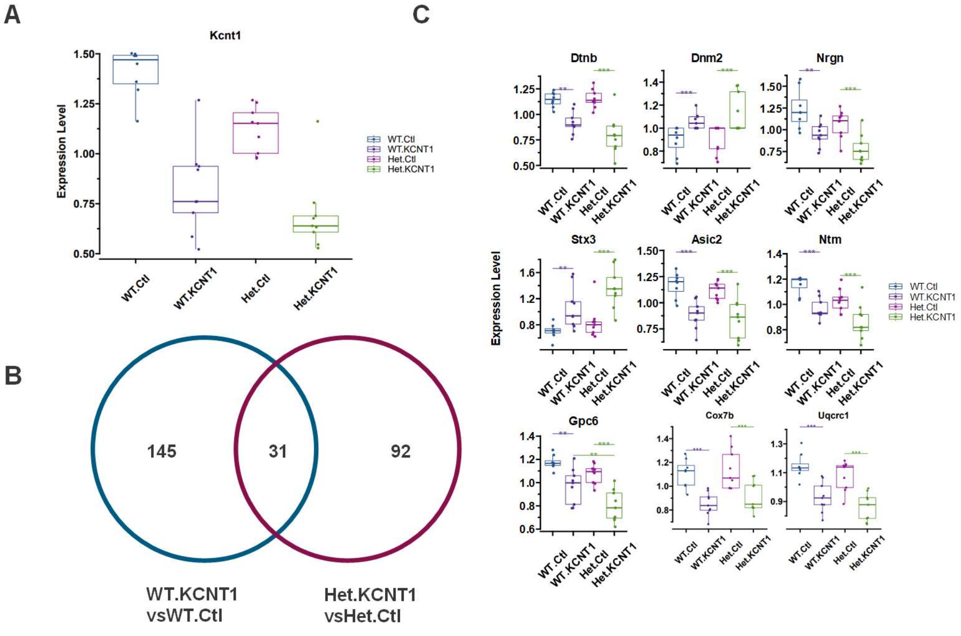

Figure S3. (A) *Kcnt1* expression in WT (control ASO and *Kcnt1* ASO treatment) and *Kcnt1*$^{R455H/+}$ (Het, control ASO and *Kcnt1* ASO treatment) mice. (B) Venn diagram of DEPs from WT and *Kcnt1*$^{R455H/+}$ mice with *Kcnt1* ASO treatment compared to control ASO. (C) DEPs annotated as synapse and mitochondrial inner membrane proteins and found in both WT and *Kcnt1*$^{R455H/+}$ mice with *Kcnt1* ASO treatment. WT.Ctl: WT mice with control ASO treatment; WT.KCNT1: WT mice with *Kcnt1* ASO treatment; Het.Ctl: mutant mice with control ASO treatment; Het.KCNT1: mutant mice with *Kcnt1* ASO treatment.

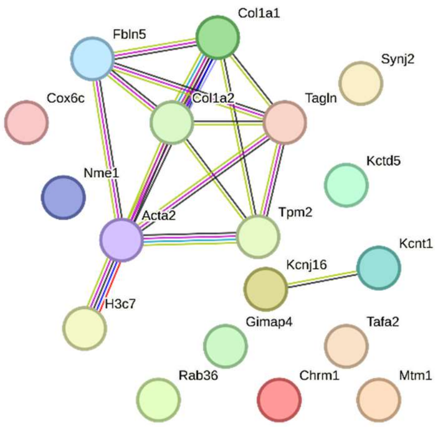

Figure S4. The string network of all the differentially expressed proteins in the *Kcnt1*$^{R455H/+}$ mice.

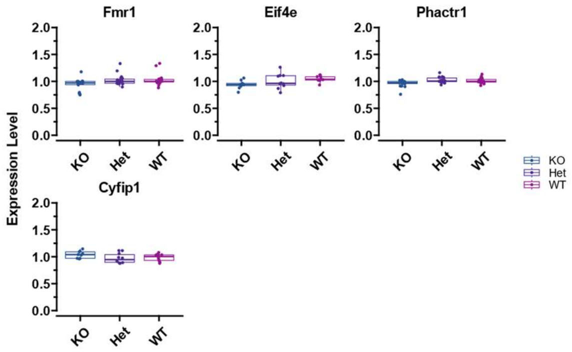

Figure S5. Proteins interacting with *Kcnt1* showed no changes in *Kcnt1*$^{R455H/+}$ (Het) and KO mice compared to WT mice.

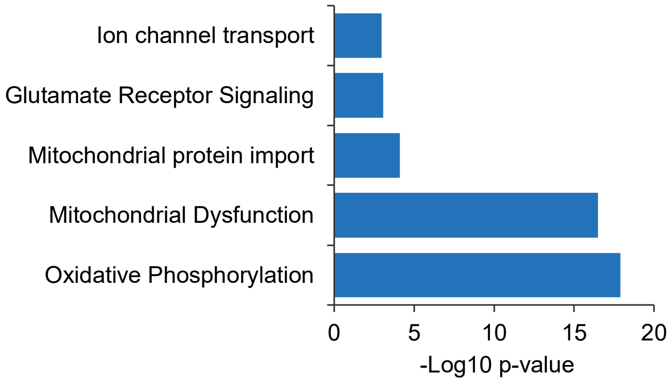

Figure S6. Top enriched pathways of proteins with significant abundance changes (P value < 0.01) in mutant mice compared to WT mice. The pathway result was generated through Ingenuity Pathway Analysis (IPA, Qiagen).

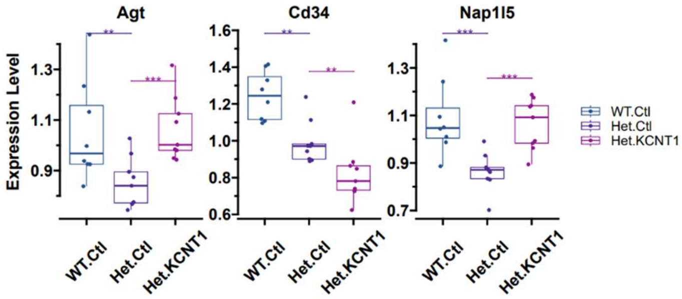

Figure S7. DEPs found in both mutant and *Kcnt1*$^{R455H/+}$ mice with *Kcnt1* ASO treatment (P value < 0.01 and $|\log_2$ fold change$| > 0.26$). WT.Ctl: WT mice with control ASO treatment; Het.Ctl: mutant mice with control ASO treatment; Het.KCNT1: mutant mice with *Kcnt1* ASO treatment.

Table S1. Up-and down-regulated genes related to GO terms of synapse and cytoplasm in Kcnt1 KO mice.

	**Up-regulated genes**	**Down-regulated genes**
**GO:0005737~cytoplasm**	ERO1B, MTCH2, PARS2, H2-K1, MPV17L2, AQP4, GHITM, MICOS10, ATP12A, NUBP1, TMEM143, TOMM22, COMTD1, FAM162A, MPC2, MPV17, SLC25A42, HMGN2, FBXW5, IGFBP2, NPY1R, SDHC, CMBL, PDYN, KCTD5, AFG3L1, ARC, SCO1, CRYZL2, GPD2, RBP1, SIDT1, TMEM126A, VDAC2, PPIH, S100A4, ATP5MD, CRABP2, ABCB7, COX15, SAR1B, FBLIM1, AKR1C18, COX7A1, FHIT, ZMPSTE24, CPLX3, APOO, FGGY, SNTG1, SLC38A2, IVNS1ABP, DMAC2L, GSTM2, ANKRD27, WFS1, ISG15, ARPC5, PAFAH2, UQCRQ, ADI1, ECHDC2, KYAT1, SERGEF, MCU, TUBA8, TINAGL1, AMD1, HSPB1, LDB3, GPT, HK2, CTSS, SLC6A4, LIMD2, CASP9, FUNDC2, CALB1, CSRP2, NNT, CTSC, CCDC51, PSD, NUDC, ANXA1, HSDL1, ANXA2, VWF, PLA2G4E, PGAM2, ANXA5, BGN, RAB3IL1, TIMM21, KCNAB3, MUP2, NME1, ACTA2, FAM210A, MYH1, ALDH1A2, ALDH1A1, DNLZ, CRH, PLEKHO1, SLC25A10, TOP1, MFGE8, SLC25A5, SHANK3, ALDH1A7, CD44, BCAT2, TAGLN, TMEM160, TUBA3A, PARL, AKAP1, SAMM50, TMEM65, NPY, DGLUCY, MAL2, SLC25A23, XDH, GABRA2, KCNIP2, TXNRD2, ATP5PB, GSR, IMMT, RPL35A, COX6C, S100B, GDAP1, GSTZ1, PNKD, NDUFAB1, SPAG1, SARDH, ATP5MPL, SLC25A35, KCNK4	RAB3B, GLTP, CDA, SRP19, CRTC3, SPARC, ISOC2B, CPNE7, SRP14, RPL6, NUDT3, CCDC91, FBXO9, MCCC2, MCCC1, ERMN, SHTN1, ADAM17, MAG, BIN1, GAPDHS, WDFY1, VOPP1, CMPK2, PADI2, UBE2V1, PHPT1, ALDH7A1, EPHA3, RAI14, H6PD, CNP, YTHDC2, NDRG1, LYPD6, MOBP, ALDH3B1, ST8SIA5, MBP, ARSG, N4BP3, MPDZ, PRPH, YARS, TOE1, WDR59, SETDB1, DNAH17, PMM1, HOMER2, ADHFE1, ANLN, WNK2, CYP2S1, CUTC, YJEFN3, HMGB3, IDE, ARHGAP5, DUSP15, CALB2, RBM3, TRIM9, RPLP2, BCAS1, SPHK2, OPALIN, PLAAT3, EMP2, PARP9, SERPINA1E, LSS, TSN, SIRT2, CLDN11, IMPACT, RPL37A, ADSSL1, CDC42EP2, ATG4C, ASPA, VAMP3, PBXIP1, WDR24, THBS4, NCALD, SERPINB1A, SLIT3, SNCB, LURAP1, DTD2, SNCA, SPAG9, PPP1R14A, KCNJ11, GABRA5, PLEKHA2, MBOAT7, EFL1, RFTN1, SRP68, ETNPPL, SUGCT, MYO1D, DHRS7, ZDHHC9, GLB1, TRMT5, TTC4, EBAG9
**GO:0045202~synapse**	SLC1A2, HSPB1, SLC6A4, CPLX3, AKAP1, EFNB2, CALB1, GLRA3, NPY, GSG1L, MAL2, DLGAP1, PSD, GABRA2, ANXA1, WFS1, ANXA5, NPY1R, CCK, UNC5C, PDYN, KCTD8, ARC, ALDH1A1, CRH, VDAC2, CBLN4, NECTIN3, SHANK3	RAB3B, C1QB, C1QA, SPARC, CHRM1, NDRG1, LYPD6, THBS4, RPL6, CALB2, TRIM9, IGSF21, GLRA2, EFNB3, RPLP2, SNCB, MPDZ, SNCA, BCAS1, PRPH, GABRA5, HOMER2, SHISA9, SIRT2, BIN1, EPHA3, C1QC, VAMP3
